# Methods of Projecting Mode Amplitude Changes on the Wavelength Axis in Order to Determine the Bending Radius on the Basis of TFBG Grating Spectra

**DOI:** 10.3390/s21227526

**Published:** 2021-11-12

**Authors:** Sławomir Cięszczyk, Damian Harasim, Ainur Ormanbekova, Krzysztof Skorupski, Martyna Wawrzyk

**Affiliations:** 1 Institute of Electronics and Information Technology, Lublin University of Technology, Nadbystrzycka 38A, 20-618 Lublin, Poland; d.harasim@pollub.pl (D.H.); k.skorupski@pollub.pl (K.S.); 2 Faculty of Information Technology, Al-Farabi Kazakh National University, Almaty 050040, Kazakhstan; ain_25@mail.ru; 3 Doctoral School, Lublin University of Technology, 20-618 Lublin, Poland; m.wawrzyk@pollub.pl

**Keywords:** TFBG sensors, demodulation algorithm, bending, signal processing

## Abstract

Tilted fibre Bragg grating (TFBG) are used as sensors to determine many quantities such as refractive index, temperature, stress, rotation and bending. The TFBG spectrum contains a lot of information and various algorithms are used for its analysis. However, most of these algorithms are dedicated to the analysis of spectral changes under the influence of the refractive index. The most popular algorithm used for this purpose is to calculate the area occupied by cladding modes. Among the remaining algorithms, there are those that use the determination of the cut-off wavelength as a surrounding refractive index (SRI) indicator. Projection on the wavelength axis can also be used to calculate the bending radius of the fibre. However, this is a more difficult task than with SRI, because the mode decay in bending is not so easy to catch. In this article, we propose a multi-step algorithm that allows to determine the impact of bending on mode leakage. At the same time, the place on the wavelength from the side of the Bragg mode and the ghost mode is determined, which represents the cladding mode radiated from the cladding under the influence of bending. The developed algorithm consists of the following operations carried out on the transmission spectrum: Fourier filtering, calculation of the cumulative value of the spectral length, low-pass filtering of the cumulative curve or its corresponding polynomial approximation, determination of the first and second derivative of the approximated curve, and projection of the second derivative of the curve on the wavelength axis. The shift of the wavelength determined in this way indirectly indicates the bending radius of the optical fibre. Based on multiple measurements, we prove that the presented algorithm provides better results when determining the bending radius compared to other algorithms adopted for this purpose and proposed for SRI measurements. Additionally, we analyse the method of determining the shift of a fragment of the spectrum using the phase of the discrete Fourier transform.

## 1. Introduction

In measurements using fibre optic sensors, in addition to the production of innovative structures, algorithms are also developed that allow, on the basis of the measured spectrum, to determine the quantity that we are interested in. New algorithms of increasing precision in determining the wavelength shift are proposed for classic Bragg gratings (FBG) [1,2,3]. New methods of analysing tilted fibre Bragg grating (TFBG) spectra [4,5,6] and tilted gratings with the plasmonic effect are also being developed [7,8]. However, these methods are dedicated to the determination of the refractive index. Other quantities that can be measured with TFBG gratings, such as temperature, stress, rotation, twist, and bending, have not been provided with dedicated algorithms. The determination of these quantities on the basis of spectra is based either on the universal algorithm of envelope of cladding modes [9], or on changes in the amplitude and shifts of individual modes [10,11].

**Figure 1:**
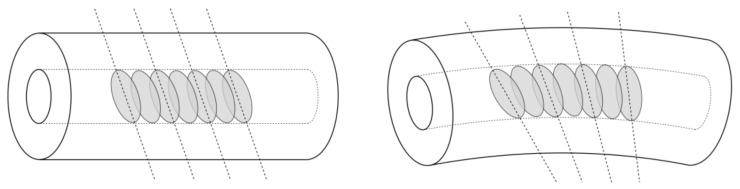
TFBG grating as a sensor for measuring the bending radius of an optical fibre.

Algorithms for determining information on the basis of data from TFBG spectral measurements use the properties of their spectra resulting from their structure. TFBGs are gratings with tilted refractive index modulation. As a result, peaks in the transmission spectrum appear corresponding to the cladding modes, which are the result of the light coupling from the core to the cladding [12]. The cladding mode comb has a different shape depending on the angle of the tilt of the refractive index. In the reflection spectrum of TFBG gratings only the Bragg mode is visible. Other modes, i.e., cladding modes, including the ghost mode, are only visible in the transmission spectrum. It is the cladding modes that are sensitive to various quantities such as the refractive index, polarisation, or just bending [13,14]. Figure 1 shows schematically the bending of the TFBG grating and the resulting changes in the angle of the polarisation planes.

Changes in the tilt angle of the polarisation planes of the TFBG grating cause changes in the transmission of the lower order modes. This mainly concerns the change of the peak amplitude. More complex systems of TFBG gratings have the possibility of determining the two-dimensional curvature [15]. Since the tilt of the refractive index changes introduces the asymmetry of the fibre and the appearance of orthogonal modes, this property can also be used for directional bend sensing [16].

Bending measurements are used especially in the monitoring of mechanical and building structures. Related to bending is the concept of curvature, which is defined as deviation from straightness. Curvature *C* is inversely proportional to the radius of the circle and can be defined as:

(1) $$C=\frac{1}{R},$$

where *R* is the radius of the circle.

**Figure 2:**
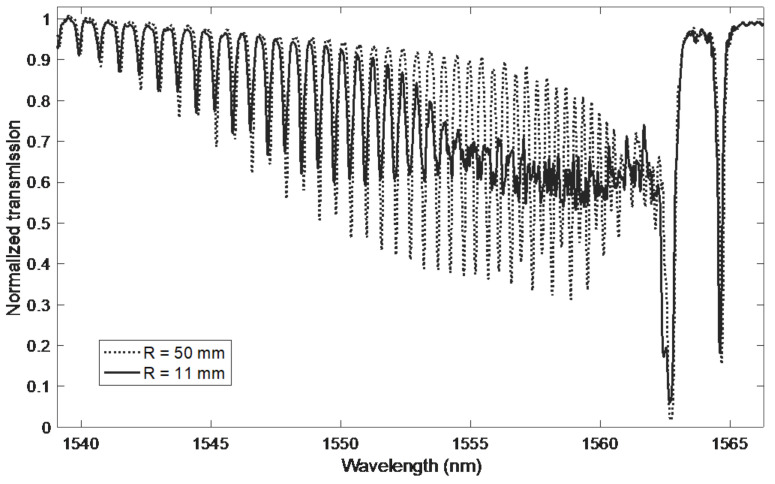
Comparison of the TFBG grating spectrum for two bending radii.

As mentioned before, the curvature changes the effective tilt angle of the plane of polarisation. Due to the increase in the curvature of the fibre, the coupling of low-order modes decreases, so the spectrum of the cladding modes lying close to the ghost mode is changing. Figure 2 shows the spectra for two bending radii of 50 and 11 mm. In the range of 1545–1563 nm, the spectra differ from each other. Particularly large differences occur in the range of 1553–1563 nm, in which the upper and lower envelope of the cladding modes are significantly reduced. After reducing the amplitude of the changes in the spectrum, some slight irregular shapes remain. Curvature changes can be detected by determining the intensity change of the modes [17]. A radius of 5 mm is indicated as distinguishable by this method [18]. The method of calculating the normalised area of cladding modes [18] can also be used to determine the curvature. As the curvature increases, successive modes change their shape. The spectrum of higher-order modes does not change. There is some similarity to the cut-off mode for surrounding refractive index (SRI) measurements. Such a demodulation method was proposed in [19], specifying that the mode leakage wavelength changes were 3 nm for a curvature change from 0 to 17 m^−1^. However, no method of precisely determining this place on the wavelength axis has been specified. It should also be noted that the main Bragg mode does not change during bending, because the core of the fibre is not stressed. The Bragg mode can therefore be used as a transmission reference peak, for example for temperature changes. Ghost mode is a composite of multiple cladding modes and is affected by bending. This influence can also be used to determine the curvature through power changes measurement [20].

The entire process of determining a given physical quantity on the basis of the analysis of the spectrum of a specific periodic structure measured with the use of optical spectrum analyser (OSA) includes, apart from the measurement itself, the algorithm used. Articles often present only a change of a given fragment of the spectrum as a result of a change in a specific physical quantity. Of course, this indicates the possibility of measurement, but only repeating the measurements and their analysis gives a full picture of the metrological properties of the method. The use of a larger fragment of the spectrum and its analysis as multidimensional data allows for algorithmic improvement of metrological properties, increased sensitivity, resolution or obtaining algorithmic selectivity for a selected measurement quantity. The proper selection of the spectrum processing method is therefore crucial in building an effective measurement system.

In order to improve the measurement properties of the system with TFBG grating for determining the bending radius, a dedicated algorithm should be developed, which will consist of several steps. A solution to this goal is the analysis of the existing algorithms for the analysis of TFBG spectra and gratings with the plasmonic effect. Then, the method should be adapted to the changes that occur in a specific case of bending the optical fibre with the tilted grating. The greatest number of algorithms was developed to determine changes in the SRI coefficient. The focus is on global methods that use the entire spectrum of cladding modes, or at least a fragment of it containing several such modes. We shall start precisely with them and their bend radius extraction capabilities. Next, we shall present an algorithm in which four main steps can be distinguished: filtering, computing the cumulative parameter, computing the derivatives of the approximated curve, and determining the characteristic wavelength. We shall also present an algorithm for determining the shift of a spectral fragment containing several modes under the influence of bending. Finally, the properties of the proposed algorithms will be compared.

## 2. Methods of TFBG Spectra Analysis

Before starting the analysis of algorithms, it is worth noting that in a large part of the publication on sensors with periodic structures, when presenting a new structure, the dependence of its spectrum on a certain quantity is presented. Although this indicates the possibility of using the structure as a sensor, it does not determine its properties. Only a sensor with an interrogation system or, in the case of spectrometric interrogation, an algorithm for extracting information from the measured spectrum allows the assessment of a given solution. Most of the algorithms for the analysis of TFBG spectra have been proposed for the determination of the SRI coefficient. Generally, these methods can be divided into two main groups. The first one analyses the spectra of a single mode, while the second one uses the spectrum analysis of a group of modes. In both cases, the analysis may consist in determining changes in the intensity of the amplitude of a single mode or a group of modes (modal strength spectroscopy [16]). For the group of modes, the most popular here is the envelope method [9], which is used not only for the measurements of the refractive index but also for the determination of the liquid level [21] and the bending curvature [18]. This method also has a more advanced version in the form of approximation of the area using a triangle mesh [22].

In the case of a single mode, the single-mode wavelength shift can also be analysed. In the domain of the wavelet transform, the collective shift of a specific group of modes can be determined [23]. For global methods, the projection of mode transmission changes to the wavelength is also used by detecting the so-called cut-off wavelength.

The most popular method of the envelope can be represented by the following formula [9]:

(2) $$A_{E}=\frac{\int_{\lambda_{\min}}^{\lambda_{\max}}[T_{up}\left(\lambda \right)-T_{low}\left(\lambda \right)]d\lambda }{\int_{\lambda_{\min}}^{\lambda_{\max}}\left[T_{up}^{ref}\left(\lambda \right)-T_{low}^{ref}\left(\lambda \right)\right]d\lambda },$$

where *T_up_* and *T_low_* are the upper and lower cladding mode envelopes. Where the denominator values are the reference values for the measurement with the largest area. A very simple parameter of the contour length represented by the following formula can also be a representation of the amplitude of the mode group [24]:

(3) $$L=\sum_{i=0}^{N-1}\left|T_{i+1}-T_{i}\right|$$

It should be noted that the contour length method uses all the spectral points in the range of the cladding modes as opposed to the envelope method, which is calculated on the basis of resonance peaks only. Another method of this type is the determination of the mean deviation from the local mean parameter, calculated as follows:

(4) $$MD=\frac{1}{N}\times \sum_{i=0}^{N-1}\left|T_{i}-{\bar{T}}_{l}\right|$$

where ${\bar{T}}_{l}$ is local average value of the transmission calculated by the averaging filter.

A slightly different approach is to calculate the integrated value of the transmission difference of two spectra in a limited spectral range [25]. We subtract the reference spectrum from the spectrum with a specific SRI, but calculate the transmission of only a few modes. This method calculates the parameter related to the amplitude of the transmission peaks, but in the range in which these amplitudes do not change. The computed integrated transmission difference changes as the spectral resonances shift with respect to the reference spectrum. This interesting method can be described as virtual differential interrogation, which algorithmically converts the mode shift into the intensity of the integrated transmission difference. The adaptability of this method consists in the possibility of selecting the spectral range to increase the sensitivity of the required SRI range. The use of this method for bending analysis will not bring positive results, because the modes do not shift in this type of measurement. Thus, no difference will be created between the modes in the integrated transmission between the two spectra.

Another group of methods are those that calculate the SRI indirectly by calculating the cut-off wavelength. Such an approach seems obvious if we analyse the mode decay under the influence of an increase in the external refractive index. The problem is, of course, how to precisely define this place on the wavelength axis, and in particular its changes. It seems that this group of methods has the potential to be more sensitive than the others. The problem here is the discrete nature of modes [26]. There is also an obvious issue related to the direction of mode fading. Increasing the SRI causes the modes to fade from the shorter wavelengths towards the ghost and Bragg modes. In the case of bending, the modes located near the ghost mode disappear first, and with larger curves the modes located further towards the shorter wavelengths. The first proposal to project changes in the amplitude of the modes on the wavelength was to calculate the derivative of the envelope formed by the cladding modes [27]. In the article by [28] it is proposed to use the curve created as a result of calculating the cumulative version of locally calculated parameters such as cumulative mean deviation from the local mean:

(5) $$CMD(k)=\frac{1}{k}\times \sum_{i=1}^{k}\left|T_{i}-{\bar{T}}_{i}\right|$$

The cut-off wavelength value is calculated at the intersection of this curve with the horizontal reference curve. This method works well for gratings with a tilt angle of 6 degrees where the modes are visibly separated from each other and the ghost mode is insignificant. In the case of gratings used for bending measurements, i.e., with small angles, type 1–2 degree, the modes near the ghost mode do not have such deep and regular shapes. Additionally, both the lower and upper envelope of cladding modes approaching the ghost mode significantly decreases. Modes do not disappear as uniformly in bending as in SRI measurements.

The mode decay projection on the cut-off wavelength determination may also be preceded by Fourier transform filtering [5]. Such a process leaves only the first harmonic of the spectrum, corresponding to the frequency of appearance of mantle mode resonances. High-frequency components related to noise and low-frequency components related to the constant component and slow-changing components are eliminated. The filtered signal is symmetrical about the transmission intensity axis. After such filtration, the authors propose to analyse the lower and upper envelope of the signal by using its peaks. Smooth curves guided through points corresponding to the maximums and minimums are approximated separately. Then the first and second derivatives are calculated for both approximated curves. The characteristic point denoting the cut-off position is the place for which the second derivative takes the value zero.

The characteristic point on the wavelength axis can be a point determined by the intersection of the transmission spectrum with a certain appropriately selected threshold value [4]. The method begins with the determination of the spectral difference between the measured spectra and the reference spectrum. The reference spectrum is the spectrum measured at an SRI greater than the cladding RI, which results in the complete disappearance of the cladding mode peaks. Subtracting this spectrum is equivalent to filtering the constant component and slowly changing components of the spectrum, which causes the spectrum to be symmetrical on the transmission intensity axis. Then, the intersection of the spectrum processed in the previous steps of the procedure with a certain horizontal reference line (threshold value) is selected as the wavelength cut-off point.

## 3. Experimental Measurement

**Figure 3:**
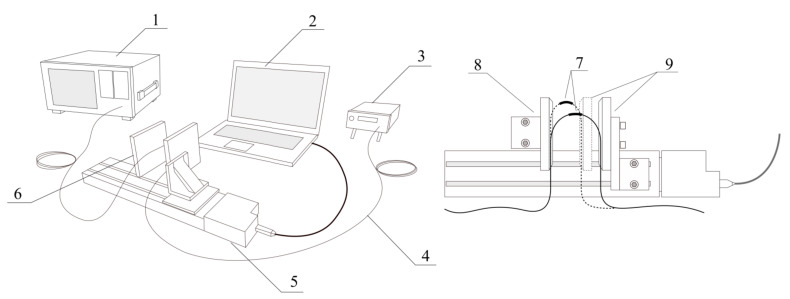
Scheme of the measuring system and the method of forcing the curvature of the fibre where: 1—optical spectrum analyser, 2—PC computer, 3—SLED broadband source, 4—single mode optical fibre, 5—automated micro-translation stage, 6—bending adjustment: 7—optical fibre section with TFBG inscribed, 8—fixed optical stage plate, 9—translation stage plate.

Experiments were performed using high-performance optical equipment. Optical spectra were measured by AQ6370D optical spectrum analyser (Yokogawa), broadband optical signal was provided by super-luminescent diode (SLED) S5FC1005S (Thorlabs Inc., Newton, NJ, USA). The light was transmitted through the TFBG inscribed in the hydrogen loaded single-mode optical fibre. All the spectra in experiments were carried out with 0.02 nm resolution and stabilized ambient temperature of 20 °C and at a constant SLD current of 250 mA. The TFBG had a 2° tilt angle and was inscribed by the phase mask method using an excimer laser (Coherent Inc., Santa Clara, CA, USA). Figure 3 shows a diagram of the measurement system with the designation of its most important elements.

The main element of the experimental system used to change the bending radius of the optical fibre in the place with the recorded mesh (grating) was an electronically controlled feed table. The optical fibre, arranged in the shape of a loop, was based on a permanently attached wall on one side. The other side was attached to a wall attached to the feed table, the shift of which was set with an accuracy of 0.001 mm using a personal computer (PC) with dedicated software. Reducing the distance between the two elements reduced the bending radius and thus increased the curvature of the TFBG recorded fibre.

**Figure 4:**
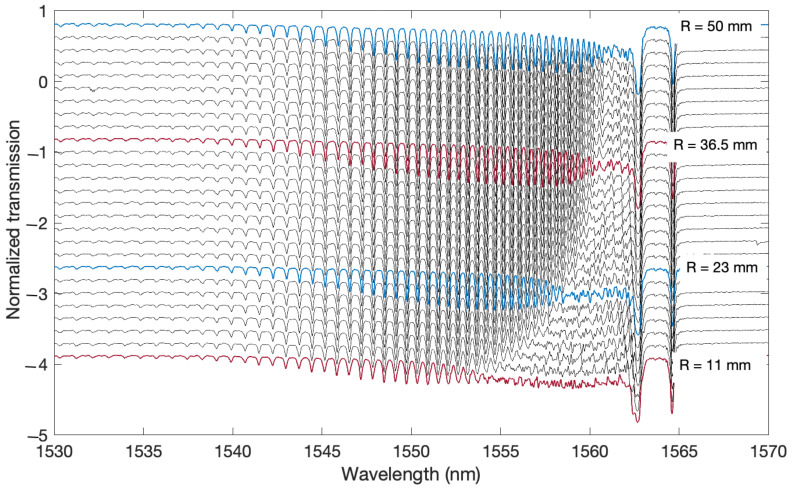
Measured TFBG transmission spectra for a few bending curvatures. Spectra for bending radii from 50 to 11 mm measured every 1.5 mm.

Figure 4 shows the spectra measured over the entire spectral range. The spectra were shifted along the amplitude axis to show the changes in the amplitude of the modes with increasing curvature. The graphs from the top were made for the radius R = 50 mm, and each subsequent graph below the bending radius was successively reduced by 1.5 mm. The smallest and lowest on the graph is the measurement for a radius of a curvature of 11 mm. Figure 4 shows the initial slow shift of the spectrum changes towards shorter wavelengths. These changes are observed at a wavelength of around 1560–1561 nm.

A greater shift occurs only for bending radii of 25–20 mm. The transmission intensity of the individual cladding modes decreases as the bend radius decreases. First, the modes closer to the ghost mode disappear. Then, successively, as the radius of curvature decreases, the amplitudes of successive modes lying further from the ghost mode decrease. The modes that lie further from a certain characteristic point on the wavelength do not undergo any changes. However, decreasing the amplitude does not completely smoothen the spectrum as it does for modes that leak due to changes in the refractive index. The shape of the remains of the decreasing amplitudes of the resonance peaks is neither regular nor smooth.

**Figure 5:**
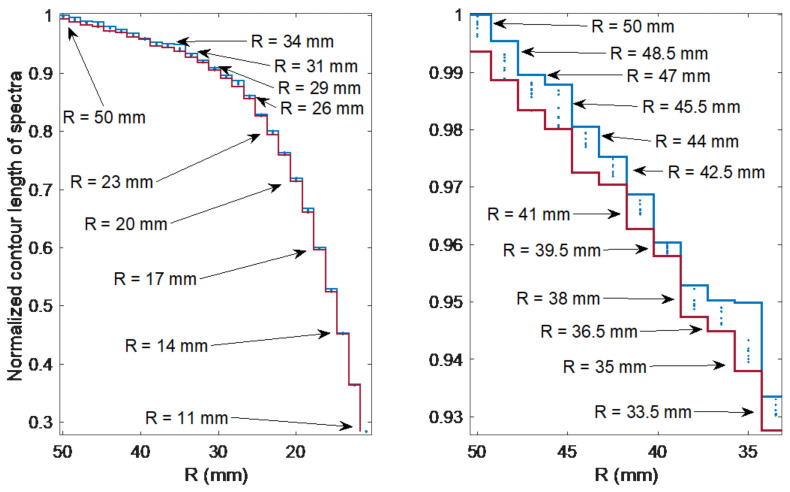
Changes in the spectral contour length under the influence of bending for 27 bending radii and for repeated measurements.

The parameter that best reflects the amplitude reduction of the individual modes is the contour length. Figure 5 shows the contour length for individual radii. For each radius, the measurement was repeated nine times. It can be noticed that for the bend radii from 50 to 34 mm it is not possible to distinguish the values of the individual bending radii. Each of the points in the figure corresponds to 1 of the 243 measurements. The lower and upper step curves correspond to the points with the largest and smallest contour length values, respectively, for nine measurements for each of the 27 radii.

## 4. Synthesis of the Algorithm for Determining the Curvature

**Figure 6:**
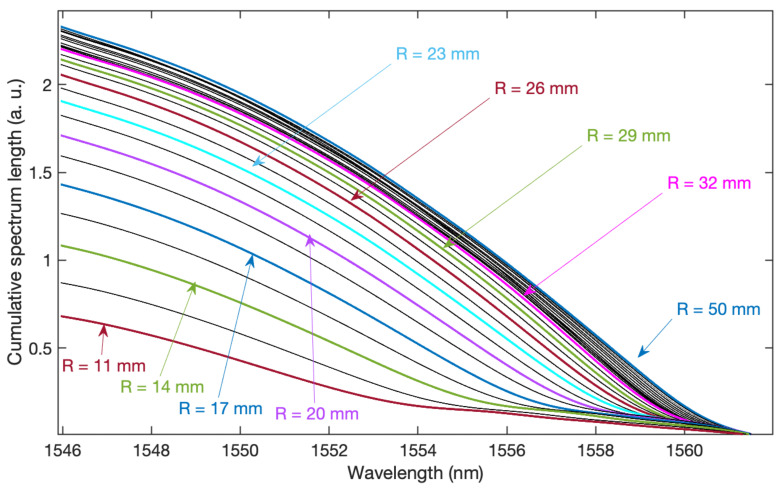
Cumulative spectrum length calculated from 1562 to 1546 nm for 27 radii from 11 to 50 mm.

**Figure 7:**
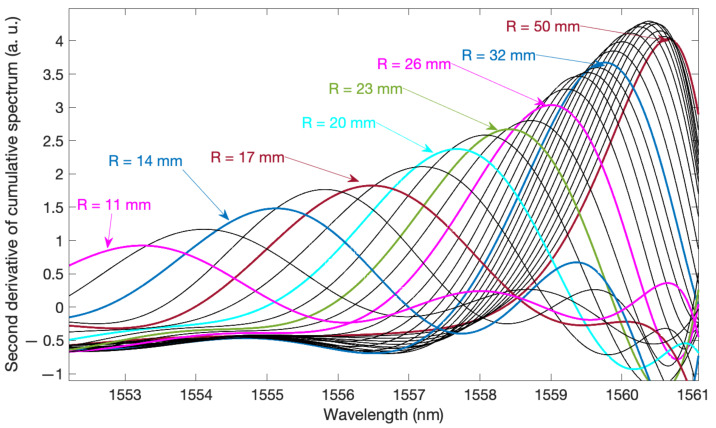
Second derivative of cumulative spectrum length.

Figure 6 shows the cumulative contour length from longer waves towards shorter waves. The calculation of the cumulative length of the spectrum was preceded by the filtering of the spectrum in the Fourier domain. The constant component and higher frequencies were eliminated, thus reducing the impact of baseline changes related to fluctuations in the radiation source and noise. The cumulative contour curve was subjected to smoothing filtration in order to eliminate the variable components related to the frequency of the individual modes. As a result of numerical experiments, the curve approximation by means of polynomials was chosen as the best method of getting rid of high-frequency components. Smoothing is necessary here because the next step of the algorithm is to calculate the first and second derivatives of the cumulative contour length curve. The graph showing the curves of the second derivative of the spectral length is shown in Figure 7. These are smooth curves that were obtained thanks to the polynomial approximation of the basic cumulative curve. Without the approximation smoothing process, the calculation of both the second and the first derivative will emphasise noise as well as undesirable irregularities. Emphasising the noise in the numerical calculation of derivatives is a phenomenon that always occurs in the analysis of real measurements, including various types of spectra. Hence the necessity of smoothing filtering, which at the same time will keep the shape of the processed curves.

The summation of the spectral length must start where the changes in the spectrum begin to occur due to the bending of the fibre. Increasing the slope of the curve means that the modes at the given wavelength have not been derived from the TFBG structure. Decreasing the bend radius moves the area of the cumulative curve slope change. However, this change is not very sharp and unambiguous, hence the need to use further mathematical operations to determine it.

**Figure 8:**
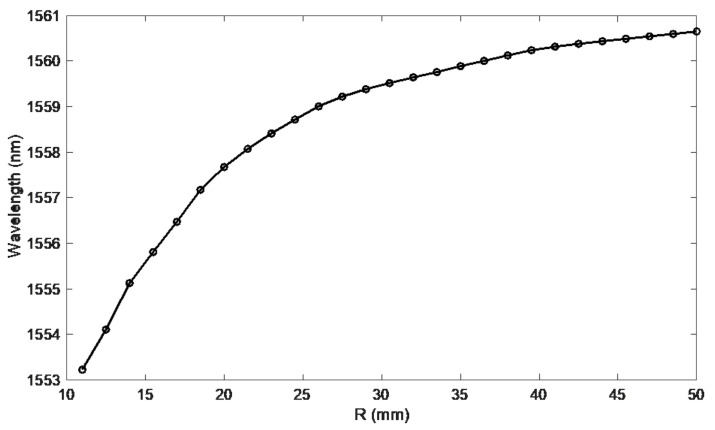
Mode leak wavelengths versus bending radius.

**Figure 9:**
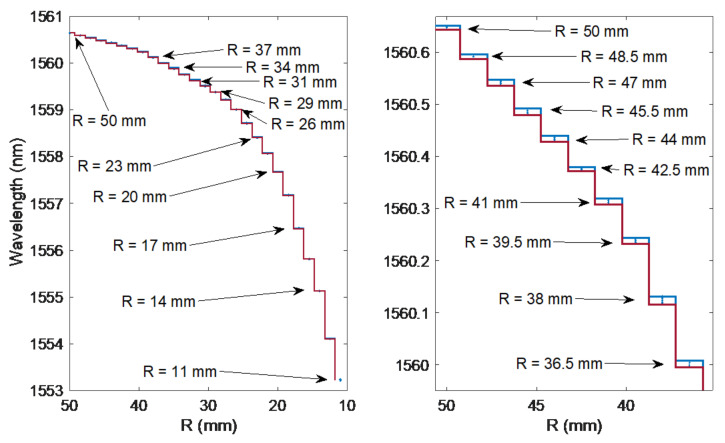
Modification of the mode leakage wavelength under the influence of the change of the bending radius of the optical fibre for 27 bending radii and nine measurements for each radius.

Figure 7 shows the calculated second derivatives of the cumulative contour length. The shape of these curves is smooth. It is also easy to see the shift of the second derivative maximum as the bend radius decreases. Even for the largest bending radii, where the spectral changes are barely noticeable, the shift of the second derivative maximum is evident. The wavelength changes for which there is a second derivative maximum are shown in Figure 8. Projecting wavelength changes related to the spectral length gives better results than a direct comparison of the spectral length itself. Comparing Figure 9 with Figure 5, one can notice significantly smaller differences for repeated measurements in the value of the calculated mode leakage wavelength than for the contour length.

**Figure 10:**
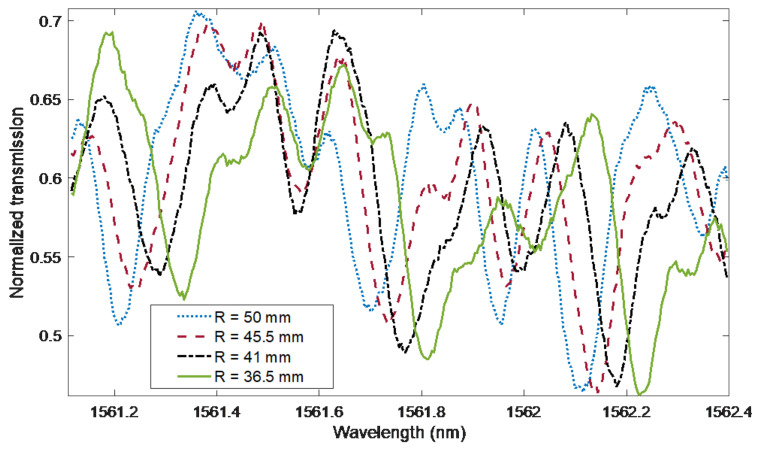
Bending effect on wavelength shift in the 1561.1 to 1562.4 nm range. Reducing the radius shifts part of the spectrum towards longer wavelengths.

Figure 10 shows a fragment of the spectrum of the cladding modes in the vicinity of the ghost mode. As can be seen, the presented modes shift towards longer wavelengths as the bend radius is reduced. These changes are already visible for radii with a value of 50 mm, i.e., in the range of curvature for which the changes in the amplitudes of the modes are small. This has the potential to combine two methods using parameters related to the wavelength axis. For small bending radii, the method of projecting changes in the contour length onto the wavelength axis, and for larger radii, the mode shift. The fragment of the spectrum in Figure 10 was multiplied by Hann apodization window. Then, a discrete Fourier transform was determined and a frequency was selected for which the phase change would be a parameter indicating the curvature changes. The signal consists of 321 points with an optical resolution of 0.004 nm, which in the Fourier domain gives a resolution of 0.7788 1/nm.

**Figure 11:**
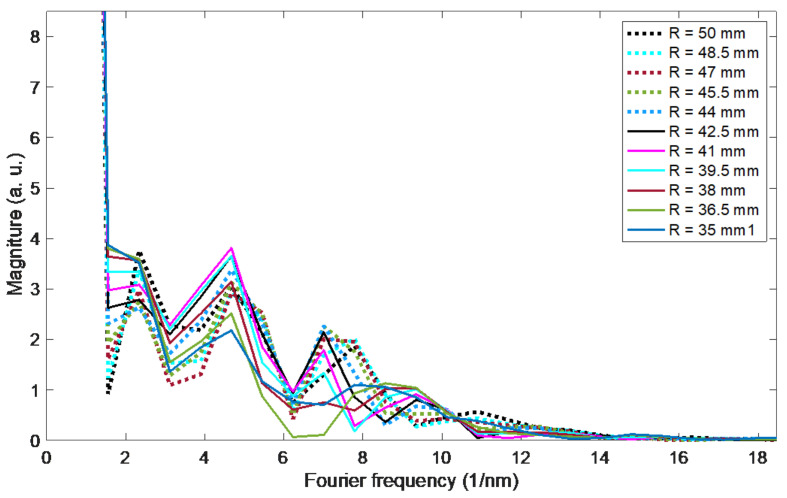
Fourier magnitude spectra corresponding to the optical spectra from Figure 10 for bending radii from 35 to 50 mm.

**Figure 12:**
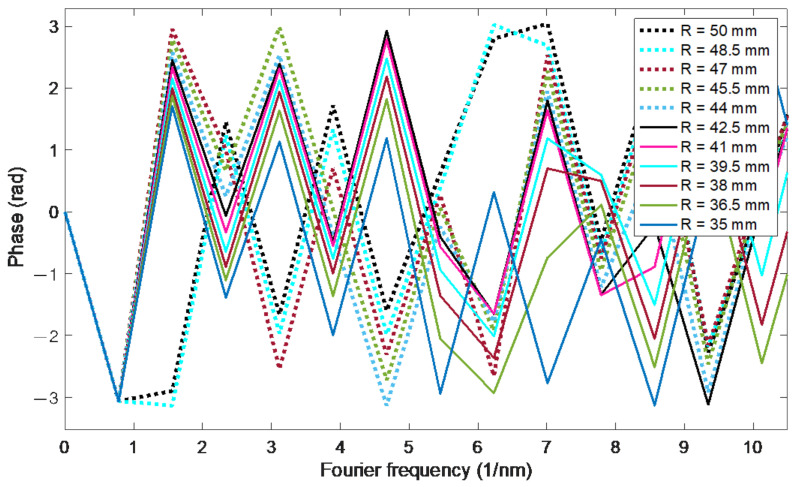
Fourier phase spectra corresponding to the optical spectra from Figure 10 for bending radii from 35 to 50 mm.

**Figure 13:**
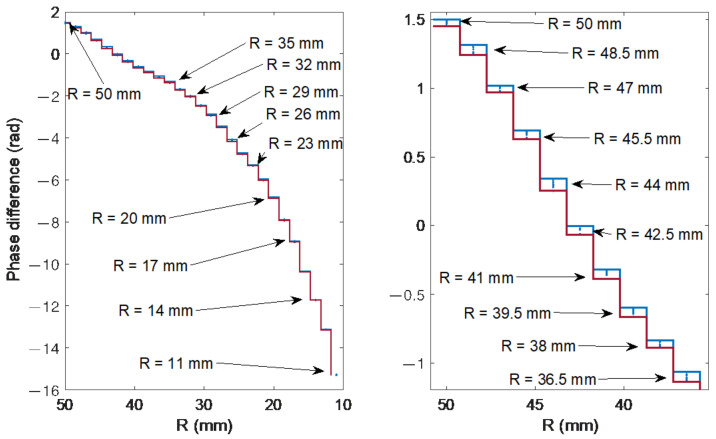
Phase changes (frequency of 2.3354 1/nm) of the spectra from Figure 10 for 27 bending radii.

The amplitude changes of the Fourier spectrum in Figure 11 are small, the high content of the constant component is characteristic. A Fourier spectrum frequency of 2.3354 1/nm was chosen for the determination of the phase (Figure 12 and Figure 13). The first three are, respectively: 0, 0.7788 and 1.5576 1/nm. The second, fifth, sixth and seventh give larger errors, a similar error rate is observed for the third. On the other hand, for further frequencies, such as e.g., 10 and 11, it is difficult to obtain unambiguous processing characteristics. This is due to the phenomenon of phase angle wrapping, which cannot be eliminated unambiguously for this frequency.

In order to compare the methods used, a nine-fold measurement of the spectrum was performed for each measured radius of curvature. Due to the nonlinear processing characteristics, the standard deviation resulting from the measurements was calculated for several measured ranges separately. The results are summarised in Table 1. There is a noticeable decrease in the value of the standard deviation along with the decrease of the bending radius. For large bending radii in the range of 35–50 mm, the algorithm that uses information about the spectrum shift in the selected range is definitely the best. For average bending radii of 20–35 mm, the algorithm using the spectral shift and the projection of the cumulative contour length on the wavelength axis is characterised by similar values of standard deviation. For small radii of 20–25 mm, the cumulative contour method has a slightly lower standard deviation. It should be noted that the simplest contour length method typically has 3–5 standard deviations as compared to the two methods using wavelength projection.

## 5. Conclusions

With spectrometric interrogation of TFBG grating, we obtain multidimensional spectral information. The use of this information depends on the selection of the appropriate algorithm and the extraction of such parameters that are correlated with the measured quantity. In the article we presented the analysis of TFBG spectrum features extraction methods in terms of their applicability to the determination of bending curvature. Most of these methods are mainly used for SRI determination. We proposed two new methods for determining the changes in TFBG spectrum under the influence of bending. We chose the simplest spectrum (contour) length algorithm as the reference method. The first proposed method is to project the cumulative changes in the contour length onto the wavelength axis. We have shown that a properly applied algorithm synthesis must be based on several component methods, of which, apart from calculating the cumulative curve, its proper filtration is also important. Filtered curves allow to calculate both the first and the second derivative. Without proper filtering, the derivative calculation causes noise to dominate the result. The wavelength at which there is a maximum of the second derivative of the cumulative contour length is an effective parameter to indicate changes in the bend curvature. The second proposed method is to determine the shift of a spectral fragment by means of a phase change of the Fourier transform. This method turned out to be the best in terms of the standard deviation of multiple measurements for large bending radii, where the amplitude changes of the transmission spectrum are relatively small. Both proposed methods allow to increase the accuracy and extend the measuring range.

## Figures and Tables

**Table 1:** Comparison of the accuracy of bending demodulation algorithms.

Range	10–20 mm	20–25 mm	25–30 mm	30–35 mm	35–40 mm	40–50 mm
Contour	0.065 mm	0.14 mm	0.26 mm	0.47 mm	0.66 mm	0.95 mm
Cumulative contour	0.019 mm	0.042 mm	0.07 mm	0.1 mm	0.15 mm	0.19 mm
FFT phase	0.025 mm	0.043 mm	0.06 mm	0.09 mm	0.11 mm	0.12 mm

## Data Availability

Not applicable.
